# Allergen immunotherapy in pregnancy

**DOI:** 10.1186/s13223-015-0096-7

**Published:** 2015-11-10

**Authors:** Paul Oykhman, Harold L. Kim, Anne K. Ellis

**Affiliations:** Department of Medicine, University of Alberta, Edmonton, AB Canada; Department of Medicine, McMaster University, Hamilton, ON Canada; Department of Medicine, Western University, London, ON Canada; Departments of Medicine and Biomedical and Molecular Sciences, Queen’s University, Kingston, ON K7L 3N6 Canada; Allergy Research Unit, Kingston General Hospital, Kingston, ON Canada

**Keywords:** Allergy, Pregnancy, Immunotherapy, Allergic rhinitis, Asthma

## Abstract

**Background:**

Allergic diseases such as asthma and allergic rhinitis constitute a significant burden of disease among women of childbearing age and those who are pregnant. Adequately managing these conditions is paramount in reducing negative fetal outcomes as well as maternal complications during pregnancy. However, the potential for harm to both the mother and fetus demands carefully balancing efficacy and safety of treatment. Allergen immunotherapy (AIT) has emerged as a relatively safe and efficacious mode of therapy in both children and adults. AIT has also been considered for use during pregnancy.

**Methods:**

A review of the literature was conducted for data regarding the safety of initiation and continuation of AIT during pregnancy as well as the effect of AIT on the development of atopy in offspring. MEDLINE and the Cochrane Library were searched for clinical trials, randomized control trials, observational studies and journal articles in English using the terms "Pregnancy" and "Immunotherapy" from 1900 to present. This yielded 4 studies (totaling 422 pregnancies receiving AIT) investigating the continuation of AIT in pregnancy, 2 (totaling 31 pregnancies receiving AIT) evaluating AIT initiation during pregnancy and 5 observing the effect of AIT on atopy in offspring.

**Results:**

No significant difference was found in the incidence of prematurity, hypertension (HTN)/proteinuria, congenital malformations or perinatal deaths between the women continued on AIT (both subcutaneous (SC) IT and sublingual (SL) IT to inhalant allergens as well as venom IT) during pregnancy and controls. Similarly, there was no significant difference in maternal or fetal complications between pregnant women initiated on AIT and controls. Among the few pregnant women (10/453 pregnancies) who experienced generalized reactions while receiving AIT, none were found to have fetal complications. Neither SCIT nor SLIT during pregnancy altered the risk of developing atopic disease in offspring.

**Conclusions:**

Based on these data, the continuation of AIT during pregnancy appears safe. Furthermore, the few data available suggest that the initiation of AIT during pregnancy might also be safe, however, more data is required for a definitive conclusion. Lastly, available studies do not show a convincing reduction in the development of atopy in offspring from the administration of AIT during pregnancy.

## Background

Asthma and allergic rhinitis are a significant cause of morbidity among women of childbearing age, including those who are pregnant. As many as 20–30 % of reproductive age women are affected by these atopic conditions [1, 2], with approximately one-third of women experiencing worsening of nasal and/or asthmatic symptoms during pregnancy [2–4]. Among women who are pregnant, 18–30 % suffer from symptoms of rhinitis and/or sinusitis [2], while up to 8.8 % are afflicted by asthma [5], and the prevalence continues to rise [5, 6]. Furthermore, up to 20 % of these women experience asthmatic exacerbations, resulting in hospitalization or even death [7]. Aside from asthma and allergic rhinitis, other allergic conditions such as Hymenoptera venom allergy also contribute to the burden of allergic disease in pregnant women [8].

Adequately controlling asthma during pregnancy is critical for the health of both the mother and fetus. Uncontrolled persistent asthma with frequent exacerbations has been associated with negative fetal outcomes such as low birthweight [7] and congenital malformations [9], as well as complications for the mother including pre-eclampsia, gestational diabetes and antepartum hemorrhage [9, 10]. Successfully controlling allergic rhinitis is also important, as uncontrolled rhinitis can not only impair sleep and overall quality of life, but can also worsen the symptoms of asthma [2, 11].

According to the National Asthma Education and Prevention Program 2004 guidelines, the conventional treatment of asthma in pregnancy consists of salbutamol and inhaled corticosteroids (budesonide in particular) plus or minus long-acting beta-2 agonists, as well as theophylline and leukotriene antagonists as adjuncts, depending on severity [12]. While a few studies have reported an increased risk to the fetus in the form of congenital malformations [13–15], the overall data supports the safety of these conventional agents, and given the substantial risk of poorly controlled asthma to the fetus, their use is generally recommended [12, 16].

Another widely used modality for the treatment of asthma and allergic rhinitis is allergen immunotherapy (AIT). Subcutaneous allergen immunotherapy (SCIT) was initially developed in the early 1900s, when it was first described by Noon that the injection of grass pollen extract reduced the symptoms of hay fever [17]. By contrast, the first randomized trial of sublingual allergen immunotherapy (SLIT) was published in 1986 [18]. Since then, SLIT has been increasingly used in Europe and more recently in the United States and Canada with approval of Oralair^®^, Grastek^®^ and Ragwitek^®^. In addition to asthma and allergic rhinitis, AIT is also commonly used for the treatment of Hymenoptera allergy. Despite the widespread use of AIT, however, few data exist regarding its safety in pregnancy.

This review outlines the existing data surrounding the initiation and continuation of AIT during pregnancy, focusing on safety for the mother and fetus, as well as future atopic outcomes for the offspring.

## Continuation of immunotherapy during pregnancy

The earliest controlled study to describe the use and safety of AIT in pregnancy was published in 1978 by Metzger et al. [19]. Metzger et al. conducted a retrospective analysis of 90 atopic women (mostly allergic rhinitis and asthma) who had undergone SCIT during one or more pregnancies, for a total of 121 pregnancies. Most of the pregnancies occurred after the initiation of SCIT, although the exact proportions were not reported. No significant difference was found in the incidence of prematurity, hypertension (HTN)/proteinuria or congenital malformations between the treated and control groups [19]. Furthermore, among the 7 generalized reactions observed in the treated group, none resulted in abnormal births [19]. These data were the earliest to suggest that SCIT was safe for continuation during pregnancy.

In a later study, Shaikh et al. retrospectively evaluated 81 atopic Indian women (again primarily allergic rhinitis and asthma) who had become pregnant one or more times during SCIT, for a total of 109 pregnancies, 102 of which were in women receiving SCIT prior to becoming pregnant [20]. As compared to the control group, the incidence of prematurity and HTN/proteinuria was actually lower in the group of women continuing SCIT during pregnancy [20]. Of the 3 patients who were observed to have a systemic reaction in response to SCIT, none were found to have any complications with the birth. Thus, as previously described by Metzger et al., continuation of SCIT during pregnancy was not associated with increased risk to the mother or fetus.

Since the first randomized control trial of SLIT in 1986, there have been more than fifty double-blind placebo controlled trials published demonstrating SLIT efficacy in children and adults. However, only one trial has thus far been conducted to evaluate its safety in pregnancy [21]. Shaikh et al. prospectively evaluated 280 atopic women (primarily allergic rhinitis and asthma) who had become pregnant one or more times, for a total of 326 pregnancies. These women were given the option of receiving either SLIT, budesonide or rescue salbutamol alone (controls). Of the 185 pregnancies receiving SLIT, 161 were continuations that were initiated prior to pregnancy. No significant difference in perinatal deaths, prematurity, HTN/proteinuria or congenital malformations were observed between the group of women continuing SLIT during pregnancy and controls [21]. Furthermore, no systemic reactions were noted in response to SLIT. This study suggests that SLIT is also a safe and viable option for continuation during pregnancy.

Most of the studies evaluating the safety of AIT in pregnancy focused on women suffering from allergic rhinitis and/or asthma. By contrast, Schwartz et al. was the first and only group to investigate the effects of venom immunotherapy (VIT) in Hymenoptera-allergic pregnant patients [8]. Schwartz et al. retrospectively analyzed 26 women with a history of prior systemic reaction to Hymenoptera venom, who had become pregnant one or more times during VIT, for a total of 43 pregnancies, 38 of which were in women already receiving maintenance doses prior to pregnancy. No significant difference in maternal/fetal complications was observed between the 38 women receiving VIT prior to pregnancy as compared to the general population [8]. No major systemic reactions were observed. Thus, in addition to AIT for allergic rhinitis and asthma, VIT may also be safe for the continuation in pregnant women.

## Initiation of immunotherapy during pregnancy

As compared to the continuation of AIT during pregnancy, data regarding the effects of initiation are scarce. In the retrospective study by Shaikh et al., of 81 atopic Indian women who had become pregnant one or more times during SCIT, only 7 of the total 109 pregnancies had been in women receiving AIT for the first time during pregnancy [20]. None of these pregnancies were found to have any maternal or fetal complications as compared to controls [20]. Of the 3 patients who had experienced systemic reactions, only one had been started on AIT during pregnancy, and no complications were observed [20].

Shaikh et al. observed similar findings in their prospective study of SLIT during pregnancy [21]. Twenty-four of the 185 pregnancies in which women received SLIT were first starts during pregnancy. Similar to the women in whom SLIT was initiated prior to pregnancy, no systemic reactions or increased maternal/fetal complications were observed as compared to controls [21].

## Immunotherapy and atopy in offspring

Although the precise mechanisms behind AIT are still being elucidated, it is known that the levels of allergen-specific IgG increase during treatment and are thought to mirror changes to the allergic response, potentially via competing with IgE for binding to allergen [22]. These allergen-specific IgG antibodies induced by AIT have been found to cross the placenta during pregnancy [23], and their levels in cord blood have been associated with reduced atopy in offspring [24].

While these correlative data suggest that AIT during pregnancy might reduce the risk of developing atopy in offspring, several studies have failed to demonstrate such benefit. In the study by Metzger et al. who evaluated 109 offspring (1/3 of which were <2 years old) of atopic mothers who received SCIT, 24.7 % exhibited allergic rhinitis or asthma [19] which was similar in incidence to the general population of children with an atopic parent. Similarly, in the retrospective study by Shaikh et al., 16 of the 60 children (age 5 months–5 years) born from mothers receiving SCIT during pregnancy had symptoms of atopic disease, which again was consistent with the incidence in children with one atopic parent [20]. Finally, in a study by Settipane et al. 38 % (15/40) of children (followed for >10 years) born from mothers who had received SCIT during pregnancy, and 45 % (17/38) of those born from untreated mothers developed asthma/allergic rhinitis, which was a non-statistically significant difference [25]. Thus, these studies suggest that continuing SCIT during pregnancy does not alter the risk of developing atopic disease in offspring.

Data surrounding the effect of SLIT on the development atopy in offspring is more limited. In the only published study using SLIT, Shaikh et al. evaluated 176 children of mothers treated with SLIT and found 24.42 % of children compared to 25.32 and 23.53 % in the budesonide and rescue salbutamol control groups, respectively, had symptoms of atopic disease, again with no difference from the incidence in the general population [21].

In a more recent retrospective survey study, Todd et al. evaluated 143 mothers with allergic rhinitis, some of which received AIT (unspecified whether SCIT or SLIT) during or prior to pregnancy, for the development of any allergic disease in offspring. Of the 277 children born to these mothers, a lower risk of allergic disease was seen in offspring from mothers receiving AIT during (OR = 0.84) or before (OR = 0.83) pregnancy as compared to atopic mothers not receiving AIT. Controlling for confounding variables (including breastfeeding, father’s allergic status etc.), however, these data were not found to be statistically significant [26].

## Conclusions

From the data described herein, it appears that the continuation of AIT during pregnancy is safe to both the mother and fetus. Indeed, according to the AAAAI/ACAAI Joint Task Force guidelines on AIT, as well as the EAACI, it is recommended that maintenance AIT may be continued during pregnancy [27, 28].

The data surrounding the initiation of AIT is limited. In each of the studies evaluating AIT during pregnancy, only a small proportion of the women were initiated on AIT during pregnancy. As such, both the AAAAI/ACAAI Joint Task Force and EAACI discourage the initiation of AIT during pregnancy until more data is available [27, 28]. A similar practice is followed in Canada.

Although there were no maternal/fetal complications observed in those few pregnant mothers who experienced generalized reactions in response to AIT, some studies have reported significant morbidity or even mortality from anaphylaxis during pregnancy [29, 30]. Thus, the benefits derived from AIT in pregnancy must be carefully balanced with the risk of anaphylaxis. Among non-pregnant patients, non-fatal or fatal reactions from subcutaneous injections of aeroallergens are rare with an incidence of <1 in one million [31]. By contrast, rates of systemic reactions with VIT have been reported to be as high as 5 % during the induction phase and 1 % during maintenance [8]. Despite these higher rates, however, the risk of VIT in pregnancy may still be justified given that treatment of sensitized women may reduce the risk of re-sting anaphylaxis from as high as 74 to 1–2 % [8, 32]. Thus, although the AAAAI/ACAAI Joint Task Force generally discourages initiation of AIT during pregnancy, it may still be considered in special high-risk scenarios such as women with prior anaphylaxis to Hymenoptera venom [27].

With respect to any potential benefit from AIT during pregnancy in reducing the development of atopy in offspring, the few studies that have been conducted thus far suggest no protective effect on the development of atopic disease. However, the protective trend seen in a recent survey study [26] highlights the need for more studies before a definitive conclusion can be generated. At this time, however, the guidelines do not endorse AIT for primary prevention of atopy in offspring [27].

Given the significant burden and growing prevalence of allergic disease among women of childbearing age and those who are pregnant, allergists will be increasingly faced with having to make treatment decisions in this population. Given the potential for harm to both the mother and fetus from treatment, balancing safety and efficacy will be paramount. AIT has emerged as a significant and efficacious mode of therapy for allergies including allergic rhinitis, asthma and Hymenoptera venom, and from the studies presented herein, may serve as a safe and viable option in pregnancy.

## Key take-home messages

- Continuation of AIT during pregnancy appears to be safe for both the mother and fetus
- Given the paucity of data regarding safety, AIT should generally not be initiated during pregnancy, with the exception of high-risk scenarios such as women with prior anaphylaxis to Hymenoptera venom where the benefits may outweigh the risks
- At this time, there is no convincing data to suggest a benefit from AIT in preventing the development of atopy in offspring, and as such, AIT should not be used exclusively for this purpose

## Abbreviations

AAAAI: American Academy of Allergy, Asthma and Immunology
ACAAI: American College of Allergy, Asthma & Immunology
AIT: allergen immunotherapy
EAACI: European Academy of Allergy and Clinical Immunology
HTN: hypertension
SC: subcutaneous
SCIT: subcutaneous immunotherapy
SL: sublingual
SLIT: sublingual immunotherapy
VIT: venom immunotherapy

## References

[1] Schatz M, Zeiger RS (1988). Diagnosis and management of rhinitis during pregnancy. Allergy Proc.

[2] Incaudo GA (2004). Diagnosis and treatment of allergic rhinitis and sinusitis during pregnancy and lactation. Clin Rev Allergy Immunol.

[3] Schatz M, Harden K, Forsythe A, Chilingar L, Hoffman C, Sperling W (1988). The course of asthma during pregnancy, post partum, and with successive pregnancies: a prospective analysis. J Allergy Clin Immunol..

[4] Kircher S, Schatz M, Long L (2002). Variables affecting asthma course during pregnancy. Ann Allergy Asthma Immunol.

[5] Kwon HL, Triche EW, Belanger K, Bracken MB (2006). The epidemiology of asthma during pregnancy: prevalence, diagnosis, and symptoms. Immunol Allergy Clin North Am..

[6] Kwon HL, Belanger K, Bracken MB (2003). Asthma prevalence among pregnant and childbearing-aged women in the United States: estimates from national health surveys. Ann Epidemiol.

[7] Murphy VE, Clifton VL, Gibson PG (2006). Asthma exacerbations during pregnancy: incidence and association with adverse pregnancy outcomes. Thorax.

[8] Schwartz HJ, Golden DB, Lockey RF (1990). Venom immunotherapy in the Hymenoptera-allergic pregnant patient. J Allergy Clin Immunol..

[9] Hodyl NA, Stark MJ, Scheil W, Grzeskowiak LE, Clifton VL (2014). Perinatal outcomes following maternal asthma and cigarette smoking during pregnancy. Eur Respir J.

[10] Enriquez R, Griffin MR, Carroll KN, Wu P, Cooper WO, Gebretsadik T (2007). Effect of maternal asthma and asthma control on pregnancy and perinatal outcomes. J Allergy Clin Immunol..

[11] Yawn B, Knudtson M (2007). Treating asthma and comorbid allergic rhinitis in pregnancy. J Am Board Fam Med..

[12] Blood I, National Heart L, National Asthma E, Prevention Program A, Pregnancy Working G (2005). NAEPP expert panel report. Managing asthma during pregnancy: recommendations for pharmacologic treatment-2004 update. J Allergy Clin Immunol..

[13] Lin S, Munsie JP, Herdt-Losavio ML, Bell E, Druschel C, Romitti PA (2008). Maternal asthma medication use and the risk of gastroschisis. Am J Epidemiol.

[14] Park-Wyllie L, Mazzotta P, Pastuszak A, Moretti ME, Beique L, Hunnisett L (2000). Birth defects after maternal exposure to corticosteroids: prospective cohort study and meta-analysis of epidemiological studies. Teratology..

[15] Blais L, Beauchesne MF, Lemiere C, Elftouh N (2009). High doses of inhaled corticosteroids during the first trimester of pregnancy and congenital malformations. J Allergy Clin Immunol..

[16] Joint Task Force on Practice Parameters AAoAA Immunology, American College of Allergy A, Immunology, Joint Council of Allergy A, Immunology (2005). Attaining optimal asthma control: a practice parameter. J Allergy Clin Immunol..

[17] Noon L (1911). Prophylactic inoculation against hayfever. Lancet..

[18] Canonica GW, Bousquet J, Casale T, Lockey RF, Baena-Cagnani CE, Pawankar R (2009). Sub-lingual immunotherapy: World Allergy Organization Position Paper 2009. Allergy.

[19] Metzger WJ, Turner E, Patterson R (1978). The safety of immunotherapy during pregnancy. J Allergy Clin Immunol..

[20] Shaikh WA (1993). A retrospective study on the safety of immunotherapy in pregnancy. Clin Exp Allergy.

[21] Shaikh WA, Shaikh SW (2012). A prospective study on the safety of sublingual immunotherapy in pregnancy. Allergy.

[22] Larche M, Akdis CA, Valenta R (2006). Immunological mechanisms of allergen-specific immunotherapy. Nat Rev Immunol.

[23] Flicker S, Marth K, Kofler H, Valenta R (2009). Placental transfer of allergen-specific IgG but not IgE from a specific immunotherapy-treated mother. J Allergy Clin Immunol..

[24] Jenmalm MC, Bjorksten B (2000). Cord blood levels of immunoglobulin G subclass antibodies to food and inhalant allergens in relation to maternal atopy and the development of atopic disease during the first 8 years of life. Clin Exp Allergy.

[25] Settipane RA, Chafee FH, Settipane GA (1988). Pollen immunotherapy during pregnancy: long-term follow-up of offsprings. Allergy Proc.

[26] Todd B, Tran QT, Lieberman P, Lieberman J (2013). The effect of allergy immunotherapy on the atopic status of offspring—a survey study. Ann Allergy Asthma Immunol.

[27] Cox L, Nelson H, Lockey R, Calabria C, Chacko T, Finegold I (2011). Allergen immunotherapy: a practice parameter third update. J Allergy Clin Immunol..

[28] Pitsios C, Demoly P, Bilo MB, Gerth van Wijk R, Pfaar O, Sturm GJ (2015). Clinical contraindications to allergen immunotherapy: an EAACI position paper. Allergy.

[29] Chaudhuri K, Gonzales J, Jesurun CA, Ambat MT, Mandal-Chaudhuri S (2008). Anaphylactic shock in pregnancy: a case study and review of the literature. Int J Obstet Anesth..

[30] Sheikh J (2007). Intrapartum anaphylaxis to penicillin in a woman with rheumatoid arthritis who had no prior penicillin allergy. Ann Allergy Asthma Immunol.

[31] Rezvani M, Bernstein DI (2007). Anaphylactic reactions during immunotherapy. Immunol Allergy Clin North Am..

[32] Reisman RE (1992). Natural history of insect sting allergy: relationship of severity of symptoms of initial sting anaphylaxis to re-sting reactions. J Allergy Clin Immunol..

